# Supplementary material for: Cloud BioLinux: pre-configured and on-demand bioinformatics computing for the genomics community
Source: BMC Bioinformatics. 2012 Mar 19;13:42. doi: 10.1186/1471-2105-13-42 (PMC3372431; doi:10.1186/1471-2105-13-42)
Supplement: Additional file 1 — Supplementary 1 Cloud BioLinux software documentation in the form of a mini, self-contained website. Users need to download and uncompress the .zip file, and open through a web browser the "index.html" file available on the main directory. (ZIP 1823 kb). [file 1471-2105-13-42-S1.ZIP › Cloud-BioLinux-Package-Documentation/docs/Preplot.html]

Bio-Linux Software Documentation Pages

Back to search form

## Preplot

|  |  |
| --- | --- |
| Name | Preplot |
| Description | **Preplot** is part of the QTL Cartographer suite of programs.  **Preplot** reformats the output of **LRmapqtl** and **Zmapqtl** so that it can be plotted by **GNUPLOT**. It requires a molecular map that was used in the analysis of the data with **LRmapqtl** and **Zmapqtl**. |
| Homepage | http://statgen.ncsu.edu/qtlcart/Preplot.php |
| Remote Documentation | http://statgen.ncsu.edu/qtlcart/Preplot.php |

Reformats the output of **LRmapqtl** and **Zmapqtl** so that it can be plotted by **GNUPLOT**.
